# “She knows me best”: a qualitative study of patient and caregiver views on the role of the primary care physician follow-up post-hospital discharge in individuals admitted with chronic obstructive pulmonary disease or congestive heart failure

**DOI:** 10.1186/s12875-021-01524-7

**Published:** 2021-09-07

**Authors:** Sarah Griffiths, Gaibrie Stephen, Tara Kiran, Karen Okrainec

**Affiliations:** 1 Department of Family and Community Medicine, St. Michael’s Hospital, Toronto, ON Canada; 2 Department of Family and Community Medicine, University of Toronto, Toronto, ON Canada; 3 MAP Centre for Urban Health Solutions, St. Michael’s Hospital, Toronto, ON Canada; 4 Institute of Health Policy, Management and Evaluation, University of Toronto, Toronto, ON Canada; 5 Toronto General Hospital Research Institute, University Health Network, Toronto, ON Canada; 6 Department of Medicine, University Health Network, Toronto, ON Canada; 7 Toronto Western Hospital, 399 Bathurst Street, 8EW-408, Toronto, ON M5T 2S8 Canada

**Keywords:** Chronic obstructive pulmonary disease, Congestive heart failure, Post-discharge, Follow-up, Primary care physician

## Abstract

**Background:**

Patients with chronic obstructive pulmonary disease (COPD) and congestive heart failure (CHF) are at high-risk of readmission after hospital discharge. There is conflicting evidence however on whether timely follow-up with a primary care provider reduces that risk. The objective of this study is to understand the perspectives of patients with COPD and CHF, and their caregivers, on the role of primary care provider follow-up after hospital discharge.

**Methods:**

A qualitative study design with semi-structured interviews was conducted among patients or their family caregivers admitted with COPD or CHF who were enrolled in a randomized controlled study at three acute care hospitals in Ontario, Canada. Participants were interviewed between December 2017 to January 2019, the majority discharged from hospital at least 30 days prior to their interview. Interviews were analyzed independently by three authors using a deductive directed content analysis, with the fourth author cross-comparing themes.

**Results:**

Interviews with 16 participants (eight patients and eight caregivers) revealed four main themes. First, participants valued visiting their primary care provider after discharge to build upon their longitudinal relationship. Second, primary care providers played a key role in coordinating care. Third, there were mixed views on the ideal time for follow-up, with many participants expressing a desire to delay follow-up to stabilize following their acute hospitalization. Fourth, the link between the post-discharge visit and preventing hospital readmissions was unclear to participants, who often self-triaged based on their symptoms when deciding on the need for emergency care.

**Conclusions:**

Patients and caregivers valued in-person follow-up with their primary care provider following discharge from hospital because of the trust established through pre-existing longitudinal relationships. Our results suggest policy makers should focus on improving rates of primary care provider attachment and systems supporting informational continuity.

**Supplementary Information:**

The online version contains supplementary material available at 10.1186/s12875-021-01524-7.

## Background

The transition to home after a hospital admission represents a vulnerable period for patients [1]. High quality transitional care is designed to support patients and their caregivers grappling with changes to medical therapies and functional status. Suboptimal transitions may lead to clinical deterioration and greater health care utilization [2]. In 2018, approximately 1 in 11 patients were readmitted to hospital within a month of their discharge, representing an estimated $1.8 billion in costs [3]. Estimates regarding which readmissions may be avoidable varies widely from 5–79% [4]. Interventions to reduce the rate of readmissions however have yielded mixed results [5–8].

Patients with congestive heart failure (CHF) have the highest 30-day readmission rates in Canada, and patients with chronic obstructive pulmonary disease (COPD) have the highest volume of readmissions [9]. Accordingly, patients with CHF and COPD are often the focus of interventions in the post-discharge period [8]. Although there is some evidence to suggest early physician follow-up may reduce the risk of 30-day readmission for those with CHF [10–12], a systematic review by Health Quality Ontario in 2017 found no difference in readmission rates with either a seven day or 30-day follow-up post-discharge for patients with CHF or COPD [13].

Despite the mixed evidence, quality improvement plans in Canada [14, 15] and financial incentives in the United States [16] have encouraged organizations to measure and improve the timeliness of primary care follow-up after hospital discharge. Timely follow-up theoretically enables close follow-up to support patient recovery at home, for example, through adjustment of medications based on symptoms and/or coordination of home, community, and specialist services to meet patient needs. Primary care physicians provide care over a lifetime taking a whole person approach and thereby help support patients with multimorbidity balancing competing medical and non-medical priorities. Studies suggest specific benefits of continuity with a familiar provider including lowered risk of death and readmission in the six-month period post-hospital discharge, independent of the timing of the initial visit [17]. One factor potentially associated with attendance at a post-discharge primary care visit is having a provider already known to the patient [18].

Although there is significant focus on improving the timeliness of primary care follow-up after discharge, there is little known about how patients and caregivers view the importance and role of the follow-up visit. Moreover, recent studies show that only a third of patients are seeing their primary care provider following discharge [19], and some research suggests that healthcare providers may have different views of the importance of the follow-up visit from patients [20–22]. To address this gap, this qualitative study was undertaken to examine the perceived importance and role of follow-up with the primary care provider post-discharge for patients admitted with COPD or CHF.

## Methods

This article was written in accordance with the standards for reporting qualitative research (SRQR) [23].

### Context and setting

This study was conducted in Ontario, Canada. Permanent residents of Ontario have access to fully insured physician and hospital services under provincial health insurance. Gaps in coverage for some could include medications, and limited public funding for home and community care. In 2017, 89.8% of Ontario residents had a primary care provider [24]. All the primary care providers of the study participants are family physicians, although other professionals, such as nurse practitioners, pharmacists, or physician specialists may see patient’s in their follow-up visit post-discharge.

### Study design

We conducted a qualitative study using semi-structured interviews to guide discussion.

### Participants

Participants were recruited from a randomized controlled trial (RCT) study population (not yet published). This study randomized individuals to a modified written discharge instruction tool compared to usual discharge instructions alone [25]. Individuals who received the instruction tool received a template written in non-medical language which outlined medication changes, follow-up plans, potential symptoms to watch out for, along with individualized plans should they occur. Study participants were patients ≥ 18 years or over, or their family caregivers, admitted with select diagnoses who were discharged from one of three acute care or rehabilitation hospitals in two cities in Ontario (Toronto and Thunder Bay). Caregivers’ perspectives were also included to acknowledge the large role caregivers often undertook in patient’s care at home in the post-discharge period, especially considering that many patients were elderly, many did not speak English as a first language, and their caregivers were often arranging and co-attending medical appointments.

A separate research team first approached all participants in the RCT about a follow-up interview. Our unpublished study protocol included different qualitative arms which sought to explore factors that influence outcomes measured in our RCT, such as follow-up with the primary care provider. Eligible participants for this qualitative study were comprised of those with either CHF or COPD, or their caregivers, who had not participated in other qualitative arms. Thirty-one participants were subsequently identified as eligible for this study; sixteen agreed to participate, two declined, and thirteen could not be reached for interview. A duration of at least one-week post-discharge was specified to allow time for participants to see their primary care provider and reflect on their experience. All participants had a primary care provider, although this was not a specified inclusion criterion. Participants who spoke a language other than English or French or with cognitive impairment were eligible if a family member also consented. All consecutive participants enrolled in the RCT who were interested in the qualitative interview were contacted for consent for this study. Enrolment stopped once thematic saturation had been met.

## Data collection

A semi-structured open-ended interview guide was developed by the authors as informed by previous studies [18] and gaps in the literature without participant input (Additional file 1): 1) perceived importance of primary care provider and other health care provider follow-up and timeliness/access; 2) role of the primary care provider in preventing readmission; 3) content explored during the visit with their primary care provider. Open-ended questions regarding each of these general domains as well as questions regarding the general transition to home after discharge and participant priorities regarding their follow-up were included. One-on-one telephone interviews were conducted by two research team members (SG, GS) from December 2017 to January 2019 and were audiotaped and transcribed for analysis. The research team members consisted of two residents in family medicine (SG, GS), a primary care physician and scientist with expertise in primary care reform on quality of care (TG) and an internal medicine physician and scientist with expertise in care transitions (KO). Research team members (SG, GS, TK, KO) had no role in participants’ clinical care. Demographic questionnaire data was collected by a separate research team led by the RCT principal investigator (KO) and was included to further understand the interview respondents’ demographics. Self-reported limited health literacy was assessed for example based on a response of “somewhat confident”, “a little confident” and “not at all confident” to the question “How confident are you in filling out medical forms by yourself?” [26].

## Data analysis

Baseline and discharge characteristics between individuals who were consented for the interview and those that did not were compared using chi-square analyses. Qualitative data was analysed using directed content analysis [27]. The transcripts were analyzed independently by three authors (SG, GS, KO), who reviewed transcripts line by line, manually, and coded them utilizing both a deductive and inductive approach, with a final author (TK) cross-comparing emergent themes. Similar initial codes were then grouped together to identify themes. At each step, disagreements were discussed during coding meetings until consensus was reached. The aim of the study being quite narrow, the study team identified saturation of themes early at 13 interviews. At this stage, the interview was modified with the input of all authors to remove or modify questions that had repeatedly produced close-ended answers. To ensure that saturation was achieved, three additional interviews were conducted, which did not contribute new findings. At this point all authors unanimously felt the main themes were saturated.

## Results

A total of 16 interviews (eight patients, eight caregivers) were conducted a mean of 57 days (SD 65 days) after discharge. Most participants were female (63%), aged 65 years or older (88%) and had an admission diagnosis of CHF (75%). In addition, 50% reported limited health literacy. Almost all participants (15/16) reported having had a follow-up with their primary care provider following discharge. Those who consented to an interview were more likely to have a physical disability (75% versus 27%, *P* = 0.01), lower level of education (44% versus 7%, *P* = 0.04) and high level of caregiver involvement (89% versus 38%, *P* = 0.05).

Our qualitative analysis identified four main themes: 1) the importance of a continuous longitudinal relationship with their primary care provider; 2) the role of the primary care provider in coordination of care; 3) preference for individualized timeline for follow-up; 4) participants self-triaging of symptoms when deciding when to seek emergency care (Table 1).

**Table 1:** Selected exemplar quotes for themes and subthemes

Themes and subthemes	Participant quotes
Theme 1: The importance of a continuous longitudinal relationship with their primary care provider	
*Sharing of experience*	[Asked why they believed it was important to follow-up with their primary care provider] “Well I wanted to let him know about the procedure and what happened.” (1146, female CHF patient aged 73 years)
*Personalized approach and relationship*	“I think that the family doctor knows the patient better [than the specialist] because they've followed them for so long and they know all of their history and they know more about it.” (5064, daughter of female COPD patient aged 79 years)
*Caring and reassurance*	“She’s cheerful and gives me hope. She's also pretty concerned also.” (2114, male CHF patient aged 58 years)
*Reinforcement and education*	[Describing the follow-up visit with the primary care provider] “He saw the report and everything because he gets a report every time she goes in there, so he knew about the different medications that she was on and what tests were done while she was in hospital, he had results of all of that, so, we kind of reviewed all that, what was good and what wasn't and, uh, you know, who was coming in since she's been home, we went over all of that so it was good and informative.” (5064, daughter of female COPD patient aged 79 years)
Theme 2: The role of the primary care provider in coordination of care	
*Information transfer*	“All of these specialists will contact with the family doc and that’s what I like about it. They’re all on the same page.” (2120, daughter of female CHF patient aged 80 years)
*Siloed care*	“Your GP is handicapped because he doesn’t have access to your system. I thought we were trying to consolidate and harmonize these information systems. It would help the GP do a much more effective job than depend on the patient to bring in a write up like “oops, I forgot my requisition.” The reliance on the patient and the family doctor is over the top and it doesn’t make sense.” (1124, female CHF patient aged 60 years)
Theme 3: Desire for individualized timeline for follow-up	“I don’t see why it needs to be sooner than that [two weeks] unless there’s a problem.” (1138, female CHF patient aged 66 years)
	“Yeah, they said a week or two and I thought within a week the doctor might not see anything different because it’s just a week and so I thought we could do two weeks. I liked that, it’s nice the way they did that.” (2120, daughter of female CHF patient aged 80 years)
Theme 4: Participants self-triaging of symptoms when deciding when to seek emergency care	“Well, I see how he is. I get that feeling that he needs to go to the hospital. I get a feeling of it, how he feels or how he looks. And usually, he doesn’t like going to the hospital, I have to force him.” (2049, son of male COPD patient aged 87 years)
	[Asked if the primary care provider could have prevented hospital admission] “No, nothing he could've done on God’s green earth.” (1146, female CHF patient aged 73 years)
	“Whatever they’re doing my mom’s doing well to the standards of her heart, diabetes and this and that. They’re doing really good and she hasn’t been back [to hospital].” (2120, daughter of female CHF patient aged 80 years)

### Continuous longitudinal relationship

Participants strongly voiced the value their primary care provider provided in their care, often in the context of having a long-standing relationship with that provider. The post-discharge visit was often discussed as a way to re-connect and share the participants’ narrative from their hospital experience. The desire to share their story persisted even if participants were aware of hospital records being transferred to their primary care provider, with one participant remarking that they felt the discharge summary did not fully explain their experience. Others often framed the importance of sharing their hospitalization experience in the context of the primary care provider being the provider who knew them best; when asked about the importance of their follow-up, one participant (1118, male CHF patient aged 74 years) stated:

“Just recognize that I have something like a 25-year history with him.”

A caregiver (5062, son of female CHF patient aged 86 years) highlighted the importance of checking with the primary care provider to ensure changes in hospital made sense for the patient:

“The family doctor knows more of her history and her medications, he can look if there has been any changes and he can look overall to see how that may affect her in the long term versus the short term.”

One participant declined a follow-up with a covering primary care provider stating:

“Yeah the secretary tried to arrange me to see another doctor within the clinic but I declined because I was concerned about my history and everything. I was more prone to seeing her [their primary care provider]” (2114, male CHF patient aged 58 years).

Participants also reported a desire to be assessed by a practitioner who was able to provide a sense of caring and reassurance. Many participants reported a positive relationship with their primary care provider and highlighted the longstanding relationship as a unique role their primary care provider had in their care. In regards to their primary care provider and their role in the post-discharge period, one participant (1146, female CHF patient aged 73 years) highlighted:

“He is a wonderful, wonderful person. Kind, caring, goes out of his way, I've been with him for many, a long time.”

Due to their primary care provider’s awareness of their individual health literacy, one participant remarked on the ability of their primary care provider to:

“Take the diagnosis and bring it down to my level” (1118, male CHF patient aged 74 years).

Other participants remarked on the ability of the primary care provider to re-iterate important pieces of information from the admission that they knew would have relevance to the individual.

### Care coordination

In a system of increasing complexity, participants often raised the importance of medical information transfer between multiple providers. Participants valued reviewing the discharge summary with their primary care provider, and often remarked that this was a key feature in their visit. When this information transfer between hospital and primary care provider was not occurring, participants would often take it upon themselves to be the conduit; bringing in their own records and sharing them with the primary care provider. Participant 1146 (female CHF patient aged 73 years) illustrates this point:

“So, I have a rheumatologist, and a respirologist and these two work together with the cardiologist, hand in hand, to figure out how they're going to help me. And my family doctor follows up and makes sure everybody gets the report of everything. I carry them with me, you know, one report the (hospital) gave me so everybody is on the same page hopefully.”

The transfer of information between various providers was also discussed as an important feature of the post-discharge visit, with the primary care provider often acting as the main coordinator. Many participants had specialist and other health care providers such as rapid response nurses, nurse practitioners, telehealth services, and physiotherapists involved in their care. When communication between these providers was well established, participants felt satisfied with their interdisciplinary care. In participants who reported a negative experience with their primary care provider, a key feature was often a lack of communication between various providers. This could be in the context of a perceived missed diagnosis due to lack of collaboration between providers, or the perceived reliance on the patient or their family caregiver to coordinate their own information transfer. One participant (2113, daughter of male CHF patient aged 85 years) expressed their concern that communication between the various care providers wasn’t occurring:

“Um, so if he could have his family doctor able to connect with each other, which, I don’t think that’s my dad’s case. You know, if they could all connect together, the heart the kidney specialist, they could always be in one place so if anything happens, they could, you know, maybe talk or discuss a medication or something.”

Many participants spoke of the pressure they believed their primary care provider faced to remain up to date on their care while grappling with a heavy patient load, paperwork, and short visits.

### Self-tailored timeline for follow-up

Participants were asked their thoughts on the appropriate timeline for follow-up for themselves post-discharge, with many expressing appointments were chosen based on convenience or a desire to trial an adjustment period, rather than a fixed timeline. Participants would often speak of this trial as a way to gather information for their primary care provider about their condition, whether this be blood pressure values, or a sense of how their recovery will progress at home. One participant (1118 male CHF patient aged 74 years) describes their rationale for waiting over a week before making their appointment:

“The reason there was a delay was in part to, uh, take some blood pressure readings and find out what the reaction was to the reduced dose of blood pressure and the increase in the furosemide. In other words, I wanted to be able to go to him with a picture of, just, how I was reacting so he had something else to put his hands around.”

Participants also felt that the appropriate time for follow-up was dependent on the particular patient context, with no one size fits all approach. One participant stated:

“Well, people are in the hospital for various reasons so, I guess, whether it’s seven days or three days or ten days depends really on the circumstances. I was comfortable with the ten-day period” (1118, male CHF patient aged 74 years).

Participants were asked if they would prefer a phone call from their primary care provider over an in-person visit, with the majority feeling that it was important to see them in person. While for many this was a function of needing to do physical exam maneuvers such as blood pressure and weight checks, one caregiver remarked he worried his mother would minimize symptoms over the phone, a sentiment that was echoed by other participants.

### Role of self-triaging symptoms

Many participants felt they were comfortable gauging the severity of their symptoms and had a good understanding of what required emergent care versus ambulatory care with their primary care provider. A caregiver (2120, daughter of female CHF patient aged 80 years) describes the process they undergo to determine if they need to bring their loved one to the emergency department:

“I would hear her breathing and say ‘ok how her breathing, is it bad enough to go to the hospital?’ I would listen to the way she talks. Does she stutter a bit? I would know the difference just from that and see the difference between this is a doctor thing or an emergency thing by looking at her and talking to her, seeing her movement.”

Some did feel a visit with the primary care provider could be helpful in the context of triaging and described their visit as a:

“First pass to ensure this wasn’t something I was making up or that this was in my head, that it was serious” (1118, male CHF patient aged 74 years).

A few participants also mentioned that in situations where their primary care provider is accessible, they do reach out to them initially as they have had experiences of having their primary care provider be able to streamline them into services. Participants echoed the general sentiment that prior training they had received on ‘reasons to go to the emergency department’ from healthcare providers either in hospital or with the primary care provider helped in this process of self-triaging and deciding when to go to an emergency department. Participants did not see the post-discharge visit as an independent factor in preventing readmissions. Many believed hospital admission was inevitable based on the severity of their health conditions, while others cited their overall care from their primary care provider as preventing them from requiring admission.

## Discussion

Our study provides the novel perspectives and experiences of patients and caregivers on the role of a follow-up visit with their primary care provider after an admission for CHF or COPD. Our findings suggest that the post-discharge visit with the primary care provider is important to the majority of participants with the fundamentally important feature being continuity and coordination of care. While many participants agreed that reviewing medications, vitals, and setting up further investigations were necessary components of their visit, the majority spoke of re-establishing their relationship with their primary care provider as the primary motivator for booking a follow-up. Participants strongly favoured in-person follow-up but there was no consensus on the optimal timing for follow-up.

Our findings are in keeping with the literature describing patients’ desire to have access to a known, trusted primary care provider [28]. Indeed, a continuous longitudinal relationship with a single primary care provider may lead to increased patient satisfaction, lower rates of preventable hospitalization [29–33] and higher rates of guideline-based care [34]. Many of our participants had home visiting non-physician health professionals, yet still had a desire to reconnect with their primary care provider for reassurance and ongoing care coordination. Future research should seek to understand how the availably of other health professionals changes the perceived necessity of a timely primary care provider follow-up [35]. Several participants in our study spoke of the role their physician plays in educating them of their new condition or reviewing their new medications, two activities have been found to improve medication adherence in patients who report a good relationship with their primary care provider [36]. As well, participants unanimously preferred an in-person visit with their primary care provider over a phone call, a finding with important implications given the growing use of virtual care during COVID-19.

Many participants believed that good primary care could prevent hospital admission however many also accepted that admissions were likely inevitable due to the severity of their health conditions. Healthcare providers have been found to be more likely to link preventable readmissions with difficulty obtaining follow-up care in the community, while premature discharge as well as problems related to housing and social supports are more commonly cited by patients and their caregivers [20]. Our participants did not see a clear link between their post-discharge follow-up specifically and re-admission risk, viewing this appointment as only one aspect of their overall continuous care. Participants’ ability to self-triage symptoms in deciding when to return to hospital may be related to the reported satisfaction and understanding with discharge instructions provided by their primary care provider regarding symptoms to watch for.

While many quality improvement initiatives are focused on improving rates of follow-up within a specific timeline of 7–14 days of discharge [37], participants in our study expressed a desire to have the timing of the visit customized to their needs and to allow an adjustment period at home. Many specifically chose to follow-up more than a week following discharge. Krumholz [37] describes a “post-hospital syndrome” of decreased psychological reserve and impaired physiology during this critical period which may explain, in part, why rates of follow-up within seven days of hospital discharge have been reported to be as low as 32% in Ontario [36] and 50% in the United States [38]. Challenges with timely access due to appointment availability, while a contributor to low rates of timely follow-up in other studies, was not one which was represented in our study. Further, participants in our study nearly unanimously attended and valued their appointment which limits the generalizability of our findings to populations without a primary care provider, those who report more limited access, and those who declined a post-discharge follow-up.

This study has some inherent limitations. The generalizability of our findings may be limited by our participant sample, who were discharged from tertiary care, often academic centers, and who all had primary care providers. Individuals in the study may have had a higher degree of caregiver involvement than many Ontarians being discharged from hospital and our small sample size does not allow us to make a distinction between themes identified by patients versus their caregivers. A more in-depth analysis such as phenomenology may have identified additional and more nuanced themes. In Ontario, COPD and CHF are both quality-based diagnoses linked to government funding, with post-discharge pathways and additional resources such as telehealth and rapid response nursing. As well, our participants were selected as part of an ongoing RCT where some of them may have received a written discharge instruction tool, the potential impact of this tool still being unknown [24]. The role these interventions may have played in the post-discharge transition is unclear. The post-discharge landscape would presumably be much different in smaller, less resourced settings. All of the study authors are clinicians who work as either hospitalists or primary care providers. While these represent different roles, the investment they share in improving the post-discharge process, either through improving pre-discharge communication, or in their role as primary care provider for those recently discharged may have influenced the conclusions drawn.

The implications for patient-centered policy from this study are many. In contrast to Ontario’s provincial quality metrics, the timeliness of the primary care provider follow-up was not a concern for our participants. Instead, our participants spoke for the need of integrated health systems to aid their primary care provider in their role as care coordinator. Most often, they spoke of the value of continuity with their primary care provider, highlighting the need for ongoing effort in supporting patient’s access to providers for whom they can have a continuous relationship with. These relationships must begin during periods of relative wellness to build the knowledge and trust that participants valued in times of stress.

## Conclusion

Our study provides new and valuable insight into the patient and caregiver perspective on the post-discharge visit with their primary care provider for those living with, or caring for those with COPD or CHF. Our findings highlight the importance of the post-discharge visit with the primary care provider to maintain continuity of care and coordinate care between various providers. Patients and caregivers preferred a customized approach to timeliness of follow-up with some explicitly preferring longer intervals; most however valued an in-person visit. Overall, participants did not see the link with their post-discharge visit and hospital readmission, but valued reconnecting with a provider they had a longstanding relationship with. This study has implications for policy makers hoping to design reforms and quality improvement targets focused on patient-centered care that considers the patient perspective on post-discharge follow-up.

## Supplementary Information

**Additional file 1.** Semi-structured interview guide

## Data Availability

The datasets generated and/or analysed during the current study are not publicly available due the ongoing nature of the randomized controlled trial but are available from the corresponding author on reasonable request.

## Abbreviations

COPD: Chronic obstructive pulmonary disease
CHF: Congestive heart failure
SRQR: Standards for reporting qualitative research
RCT: Randomized controlled trial
