# Supplementary material for: “She knows me best”: a qualitative study of patient and caregiver views on the role of the primary care physician follow-up post-hospital discharge in individuals admitted with chronic obstructive pulmonary disease or congestive heart failure
Source: BMC Fam Pract. 2021 Sep 7;22:176. doi: 10.1186/s12875-021-01524-7 (PMC8421240; doi:10.1186/s12875-021-01524-7)
Supplement: Supplementary file 1 — Additional file 1. Semi-structured interview guide [file 12875_2021_1524_MOESM1_ESM.docx]

Additional File 1. Semi-structured interview guide

Introductory Script

Thank you very much for agreeing to participate in this interview. We are interviewing you to better understand what patients think about seeing their family doctor after they leave the hospital and how to improve this visit. There are no right or wrong answers to any of our questions, we are interested in your own experience.

Participation in this study is voluntary and your decision to participate, or not participate, will not affect the care you receive. The interview should take between thirty minutes to one hour depending on how much information you would like to share. With your permission, I would like to audio record the interview because I do not want to miss any of your comments. All responses will be kept confidential. This means your interview responses will only be shared with research team members and we will not be able to identify you as the respondent. You may decline to answer any question or stop the interview at any time and for any reason.

Are there any questions about what I have just explained? May I turn on the digital recorder?

Opening

Tell me a little about your most recent admission to hospital? What was the reason for admission and what led you into the hospital?

*Perceived importance of family doctor follow-up*

1. How important do you think it is for you to see your family doctor after discharge from hospital? (Prompts: What makes it important or not important? What is difficult about leaving the hospital and going back home and how can your family doctor help you in that time?)
2. How soon after discharge do you think you should see your family doctor? (Prompt: Do you think an in-person appointment is needed or could issues be dealt with in another way like a phone call?)
3. Aside from the family doctor, what other doctors or health care professionals after discharge? (Prompts: How was your appointment with your family doctor different than the appointment with the specialist? Were there any other medical professionals you spoke to after you left the hospital that you found useful to talk to? If so, why? How do you think the various medical professionals involved in your care work together?)

*Family doctor and the role in preventing re-admission*

1. Do you think your family doctor could help you from having to be readmitted to the hospital? If so, how? If not, why? (OR: do you think this admission could have been prevented by your family doctor?)
2. How easy is it to see your family doctor after hours?
3. Has your family doctor ever discussed the signs and symptoms that you should go to the ER for?
4. When you are feeling worse, how do you make the decision to try to reach your family doctor versus going to the emergency?

*Experience at the family doctor visit*

- - - 1. Tell me about your experience making the appointment with your family doctor?
      2. Can you walk me through what happened at your first appointment with your family doctor? (Prompts: Were there any changes in your medications? If so, did your family doctor go through them with you and did you find this helpful? We have heard from people that there is a lot of new information when you are discharged from the hospital. Was there any confusion around your discharge instructions?)
      3. What was your overall experience at your visit? (Prompt: Did you feel like you had enough time to address your concerns?)
      4. What is your relationship like with your family doctor? (Prompt: Do you feel heard/listened to by your family doctor?)
      5. Is your family doctor part of a Family Health Team (provide some explanation as to what this is)? If so, do you find this beneficial for your care?

Closing

Was there anything else you want to mention regarding your family doctor or their appointment with their family doctor that we did not cover today that you think is important for their health after being admitted to hospital?
